# Emerging insights to lung cancer drug resistance

**DOI:** 10.20517/cdr.2022.61

**Published:** 2022-06-21

**Authors:** Chunxia Su

**Affiliations:** Department of Oncology, Shanghai Pulmonary Hospital & Thoracic Cancer Institute, Tongji University School of Medicine, Shanghai 200433, China.

**Keywords:** Non-small cell lung cancer, drug resistance, targeted therapy, immunotherapy

## Abstract

Lung cancer remains the malignant tumor with the highest morbidity and mortality in China, with non-small cell lung cancer (NSCLC) accounting for 80%-85% of cases. Nowadays, the treatment pattern of NSCLC has evolved toward precision management with the development of molecular targeted therapy and immunotherapy. However, the median overall survival for patients with metastatic NSCLC, unfortunately, remains less than three years. Drug resistance is the bottleneck to preventing drugs from playing a further role, and the mechanistic study of drug resistance is the prerequisite for new regimen development. This Special Issue pays special attention to drug resistance in the treatment of NSCLC. We received and published several excellent articles regarding this topic. We hope that, through this Special Issue, we can have a deep understanding of the existing problems, the underlying mechanism, and the future solutions and that the publication of this Special Issue can bring some inspiration to readers.

## INTRODUCTION

According to the most recent statistics from the National Cancer Center of China^[1]^, lung cancer remains the most common malignant tumor as well as the first leading cause of cancer-related deaths in China, with an estimated 0.828 million new cases and 0.657 million deaths per year, placing a heavy burden to public health and economy development. Approximately 80%-85% of lung cancers are classified pathologically as non-small cell lung cancer (NSCLC)^[2]^. With substantial improvements in understanding of tumor biology, genomic mutational landscapes, cancer immunology, and tumor microenvironment, the treatment pattern of NSCLC has evolved toward precision management^[3]^. Molecular targeted therapy based on different driver oncogenes and immune checkpoint inhibitors (ICIs) based on programmed cell death ligand 1(PD-L1) expression are the 2 major branches of personalized treatment paradigm, which have brought great survival benefits to patients with oncogene-addicted and non-oncogene-addicted cancers, respectively^[4]^. However, the median overall survival for those presenting with metastatic NSCLC, unfortunately, remains less than 3 years^[4]^. Drug resistance is always the bottleneck to preventing drugs or new regimens from playing a further role. This Special Issue pays special attention to drug resistance in the treatment of NSCLC. We hope that, through this issue, we can have a deep understanding of the existing problems, the underlying mechanism, and the future solutions and that the publication of this Special Issue can bring some inspiration to readers. 

## MAIN TEXT

### Resistance mechanism for targeted therapy

#### EGFR

The activating mutation of epidermal growth factor receptor (EGFR) is highly common in Asian patients with NSCLC, accounting for 40%-50% of cases^[5]^. Despite the significant benefits of EGFR-tyrosine kinase inhibitors (EGFR-TKIs), patients with EGFR activating mutation inevitably develop drug resistance within a certain treatment period. Nowadays, many studies focus on resistance mechanisms to third-generation EGFR-TKIs, as they have been recommended as the preferred first-line treatment for advanced NSCLC patients with EGFR activating mutation. Numerous resistance mechanisms have been identified^[6-8]^, which can be broadly classified into two groups: (1) The first includes EGFR-dependent mechanisms, mainly referring to secondary or tertiary mutations, such as C797S, G724, L792, L718, and G719; (2) the second includes EGFR-independent mechanisms, including activation of downstream signaling pathways such as human epidermal growth factor receptor 3 (HER3), anexelekto (AXL), fibroblast growth factor receptor (FGFR) signaling pathways, *etc*.; acquisition of other potentially targetable oncogenic drivers such as mutations in phosphatidylinositol‐3‐kinase catalytic α (PIK3CA), Kirsten rat sarcoma (KRAS), BRAF V600E, anaplastic lymphoma kinase (ALK) fusions, MET amplification, *etc*.; and histological transformation to small-cell or squamous carcinoma.

Recently, some new mechanisms have been revealed. For example, Kashima *et al*. identified CD74 upregulation as a novel mechanism of resistance to osimertinib by applying single-cell analyses to cell models^[9]^. Nilsson *et al*. found that the activation of yes-associated protein (YAP) and forkhead box protein M1 (FOXM1) could mediate epithelial-to-mesenchymal transition (EMT)-associated EGFR-TKIs resistance^[10]^. These mechanistic studies have promoted some potential strategies to overcome EGFR-TKIs resistance. The allosteric inhibitor of src homology 2 domain-containing phosphatases (SHP2), a blockade to receptor tyrosine kinases (RTK) signaling, was recently shown to be a therapeutic strategy in acquired EGFR-TKIs-resistant NSCLC at the preclinical stage^[11]^. The combination of osimertinib and a novel AXL inhibitor ONO-7475 showed remarkable activity on osimertinib-resistant tumor cells *in vitro* and *in vivo*^[12]^. Zhu *et al*. identified a novel connection between osimertinib and c-Myc and further demonstrated targeting c-Myc as a potential strategy to overcome osimertinib acquired resistance in cell lines^[13]^. However, these therapeutic strategies need to be further validated in clinical trials and more research is welcomed.

#### KRAS

The KRAS gene is the most frequently mutated oncogene in human cancer, accounting for approximately 30% of NSCLC^[14]^. However, because RAS protein has a picomolar affinity for GTP and lacks known allosteric regulatory sites, KRAS had long been considered “undruggable” despite 40 years of sustained efforts. In 2013, the breakthrough by Ostrem *et al*. of small molecules that covalently bind to the acquired cysteine residue within the switch II region in KRAS^G12C^ opened the door to therapy targeting KRAS^[15]^. In recent years, a series of clinical trials have evidenced the remarkable antitumor activity of KRAS^G12C^ inhibitors such as AMG510 and MRTX849, with a response rate of 30%-40%^[16,17]^. However, drug resistance also has limited survival benefits for most patients, with a median progression-free survival of 6.3 months. Similarly, the resistance mechanisms of KRAS^G12C^ inhibitors are divided into the KRAS-dependent and -independent ways. The reported acquired KRAS alterations^[18,19]^ include G12D/R/V/W, G13D, Q61H, R68S, H95D/Q/R, Y96C, Y96D, Y96S, and high-level amplification of the KRAS^G12C^ allele, whereas KRAS-independent mechanisms^[20,21]^ also involve bypass signaling activation, such as upstream RTK regulators (EGFR, HER2, FGFR, and SHP2), direct mediators of KRAS activation (AURKA), and/or effectors of mitogen-activated protein kinase (MAPK) and PI3K pathways (MYC and mTOR); acquired alterations of other oncogenes, such as NRAS, BRAF, MAP2K1, RET, ALK, FGFR3, CDKN2A, PTEN, *etc*.; and histological transformation to squamous cell carcinoma. In addition, impaired antitumor immunity could confer resistance to KRAS^G12C^ inhibition independent of the above mechanisms, which might be attributed to the partial antitumor efficacy of KRAS^G12C^ inhibitors derived from the activation of an immune response, as KRAS^G12C^ inhibition could induce a pro-inflammatory tumor microenvironment (TME) and prime antigen-presenting cells and cytotoxic T cells^[22]^. Therefore, the feasibility and efficacy of the combination of KRAS^G12C^ inhibitor with immunotherapy are worthy of further studies. 

Together, although drugs targeting KRAS were first developed less than 10 years ago and only two kinds of targeted drugs have entered the clinic and are both under phase III clinical trials now, the mechanisms of drug resistance have been studied to develop effective therapeutic strategies better. However, more relevant clinical data are needed in the near future when the drugs are widely used in clinical practice.

### Resistance mechanism for ICIs-based therapy

Immune checkpoint inhibitors (ICIs) targeting programmed cell death 1 (PD-1) and its ligand (PD-L1) have revolutionized the treatment scenario for patients with NSCLC. Albeit with unprecedented improved long-term survival in selected patients, ICIs failed to achieve a high response rate for the whole population. Because of the specific kinetics and patterns of response to ICIs, their resistance mechanisms are much different from those of chemotherapy or targeted therapy. In 2020, the Society for Immunotherapy of Cancer published an expert consensus on clinical definitions for resistance to ICIs in three distinct scenarios: primary resistance, secondary resistance, and progression after treatment discontinuation^[23]^.

Primary resistance is defined as evidence of disease progression after receiving at least 6 weeks (2 cycles) of exposure to ICIs but no more than 6 months. This type of resistance is related to the inability of the immune system to activate an appropriate immune response to malignant neoplasm, which can be due to any impaired functions in the cancer-immunity cycle^[24]^, including cancer antigen release and presentation; T-cell priming, activation, trafficking, migration, and infiltration; or recognition and killing of cancer cells by T cells. Factors contributing to this resistance involve tumor-intrinsic and tumor-extrinsic mechanisms. Tumor-intrinsic mechanisms refer to the cancer cells enabling the suppression of the immune response by modulating their genome, transcriptome, and proteome. Some gene alterations^[25,26]^ [loss of phosphatase and tensin homolog (PTEN), the mutation in Liver kinase B1 (LKB1), TP53, EGFR, ALK, *etc*.], and oncogenic signaling pathways^[27]^ (WNT/β-catenin, MYC, MAPK, JAK/STAT3, *etc*.) decrease T-cell infiltration. LKB1-mutation also results in the recruitment of suppressive myeloid cells, and the inhibition of granulocytic myeloid-derived suppressor cells (G-MDSCs) via all-trans-retinoic acid could overcome the resistance of PD-1 blockade in LKB1-deficient murine NSCLC^[28]^. Yang *et al*. discovered that USP12 downregulation fostered an immunosuppressive microenvironment with increased macrophage recruitment and reduced T-cell activation via NF-κB hyperactivation in tumor cells^[29]^. Recently, Wennerberg *et al*. described the expression of mono-adenosine 5’-diphosphate-ribosyltransferase 1 (ART1) on tumor cell-mediated cell death of P2X7R+ CD8 T cells as a novel mechanism of immune resistance in NSCLC and provided preclinical evidence that antibody-mediated targeting of ART1 could improve tumor control^[30]^. Tumor-extrinsic factors are those components beyond cancer cells that contribute to suppressive TME. For example, the overexpression of vascular endothelial growth factor (VEGF) on endothelial cells promotes MDSC infiltration and decreases T-cell infiltration^[31]^. Cancer-associated fibroblasts (CAF), one of the most abundant components of TME, could also create an immune-suppressive TME by increasing the infiltration of MDSCs and tumor-associated macrophages, promoting the polarization of macrophages and reducing proliferation and antitumor activity of CD8^+^ T cells and natural killer cells^[32]^. Recently, Horton* et al*. reported that abnormal differentiation of CD8^+^ T cell during priming mediated ICIs resistance in T cell-infiltrated NSCLC^[33]^.

Acquired resistance is defined as evidence of disease progression after experiencing clinical benefit (either objective response or stable disease lasting 6 months or greater). As the name implies, this type of resistance is “acquired” through the adaption of tumor cells to the host immune system, reflecting the evolution of the tumor as a malignant organism under selection pressure from therapeutic drugs. Early research^[34]^ suggested that tumor cell-autonomous defects in interferon (IFN) signaling through JAK1/2 inactivating mutations or HLA class I antigen processing through mutation or loss of beta-2-microglobulin (B2M) mediated acquired resistance to ICIs. Neoantigen depletion was also identified to lead to subsequent immune evasion in NSCLC^[35]^. The upregulation of additional coinhibitory receptors^[36]^, such as LAG-3, TIM-3, TIGIT, VISTA, *etc*., could also abrogate the effect of PD-1/PD-L1 blockade. In addition, acquired resistance to ICIs could also arise upon selection for new oncogenic variants that mediate T-cell exclusion, including loss of PTEN and WNT/β-catenin activation^[34]^. Recently, the upregulation of CD38 induced by ATRA and IFN-β was found to mediate acquired resistance by suppressing cytotoxic T-cell proliferation, antitumor cytokine secretion, and killing capability via adenosine receptor signaling^[37]^.

Considering the special context that disease progression occurs after discontinuation for adjuvant/neoadjuvant immunotherapy with a fixed duration, or for the metastatic setting either secondary to toxicity or after achieving maximal benefits, the conception of “progression after treatment discontinuation” was proposed. In terms of mechanism, this type of resistance can be mediated by the elements from either “primary resistance” or “acquired resistance”. The early recurrence after discontinuation of adjuvant therapy may resemble primary resistance, whereas late recurrence after adjuvant therapy discontinuation and initial disease control may resemble acquired resistance. However, up to now, studies focused on resistance mechanisms specifically in these settings are lacking.

Taken together, resistance mechanisms to ICIs are much different from those to chemotherapy or targeted therapy, in which tumor-intrinsic and tumor-extrinsic factors both need to be considered. Although there are many barriers to investigating resistance mechanisms to immunotherapy, including difficulty acquiring optimal tumor samples for analyses and the absence of routine and effective tools to comprehensively interrogate alterations in the tumor, host, and/or microenvironment, more in-depth studies are needed to better clarify the underlying molecular mechanism and develop corresponding rational therapeutic strategies to overcome resistance. Of note, there is a lack of studies on resistance mechanisms to combinations of ICIs and chemotherapy, since the synergistic antitumor activity leaves it difficult to distinguish which component is the driver of response.

In this Special Issue, some new factors are reported to contribute to ICI resistance. Wu *et al*. found that circRNA hsa_circ_0020714 was related to a poor prognosis of anti-PD-1 immunotherapy in NSCLC, and a mechanistic study suggested that hsa_circ_0020714 functioned as an endogenous miR-30a-5p sponge to enhance SOX4 expression, thereby promoting immune evasion and anti-PD-1 resistance in NSCLC patients^[38]^. Their study raised the important role of circRNA in immune resistance and provided a potential targeted pathway “hsa_circ_0020714/miR-30a-5p/SOX4” to overcome resistance to anti-PD-1 therapy. The role of tumor-derived exosomes in tumor response to immunotherapy was recapitulated by Wu *et al*., and they pointed out that tumor-derived exosomes should be studied and manipulated to provide clinical benefits and improve the clinical management of lung cancer^[39]^. Li *et al*. reviewed the resistance mechanism to ICIs, especially in KRAS-mutant NSCLC, with a critical focus on metabolism remodeling mediated by the oncogenic KRAS pathway, and they argued that these alternated metabolic pathways could be promising approaches to overcome immunotherapy resistance^[40]^. From the present clinical practice, Yu *et al*. discovered that the use of ICIs plus chemotherapy and/or anti-angiogenesis therapy correlated with better survival after resistance to previous ICI treatment, which provides a therapeutic option for patients with resistance to ICIs before more reliable strategies enter the clinic^[41]^. More research on lung cancer drug resistance is needed to improve patient survival in the future.

## DECLARATIONS

### Copyright

© The Author(s) 2022.
